# The *Proust Machine*: What a Public Science Event Tells Us About Autobiographical Memory and the Five Senses

**DOI:** 10.3389/fpsyg.2020.623910

**Published:** 2021-01-20

**Authors:** Alexandra Ernst, Julie M. F. Bertrand, Virginie Voltzenlogel, Céline Souchay, Christopher J. A. Moulin

**Affiliations:** ^1^ Laboratoire de Psychopathologie et de Neuropsychologie (EA 2027), Université Paris 8 Vincennes, Saint-Denis, France; ^2^ Laboratoire de Psychologie (EA 3188), Université de Franche-Comté, Besançon, France; ^3^ CERPPS, Université de Toulouse II – Jean Jaurès, Toulouse, France; ^4^ LPNC (CNRS UMR 5015), Université Grenoble Alpes, Grenoble, France

**Keywords:** autobiographical memory, cuing techniques, reminiscence bump, self, psychopathology, olfaction, participatory science

## Abstract

Our senses are constantly stimulated in our daily lives but we have only a limited understanding of how they affect our cognitive processes and, especially, our autobiographical memory. Capitalizing on a public science event, we conducted the first empirical study that aimed to compare the relative influence of the five senses on the access, temporal distribution, and phenomenological characteristics of autobiographical memories in a sample of about 400 participants. We found that the access and the phenomenological features of memories varied as a function of the type of sensory cues, but not their temporal distribution. With regard to their influence on autobiographical memory, an overlap between some senses was found, with on one hand, olfaction and taste and, on the other, vision, audition, and touch. We discuss these findings in the light of theories of perception, memory, and the self, and consider methodological implications of the sensory cuing technique in memory research, as well as clinical implications for research in psychopathological and neuropsychological populations.

## Introduction

[…] smell and taste still remain for a long time, like souls, remembering, waiting, hoping, upon the ruins of all the rest, bearing without giving way, on their almost impalpable droplet, the immense edifice of memory.

—Marcel Proust, *Du côté de chez Swann*, 1913

Since the late 1980s, there has been growing interest in the concept of autobiographical memory, the study of memory for one’s own life experiences. The interest in this domain arguably grew out of the real world memory debate (e.g., Banaji and Crowder, 1989), and owes a debt to early attempts to understand memory from a naturalistic, ecologically valid perspective. As such, studies of autobiographical memory lend themselves to large scale survey and public participation events, with recent examples being large-scale on-line questionnaires about autobiographical memory (e.g., Janssen et al., 2011) or studies examining memories cued by Beatles songs (Spivack et al., 2019), and data collected at Science Festivals (e.g., Alleyne and Carter, 2008). Furthermore, an often overlooked aspect of the open science movement is the idea of sharing results, methods, and procedures, implicating the public in all aspects of scientific inquiry. In this vein, we were motivated to share what was known about autobiographical memory in a participatory science event, *the Proust Machine*, but also to harness the event to examine the particular relationships between the various types of the cue, and the resultant autobiographical memories.

Marcel Proust’s inspiration for numerous studies looking at olfaction and memory is clear. However, despite Proust’s intuition of the fundamental influence of the senses on memory, little empirical research in the cognitive sciences has tackled this issue in humans, yet this topic is critical to understand the richness of autobiographical memory functioning in everyday life. Who has never experienced once in his/her life the feeling of being suddenly transported back in the past, reliving vivid and rich memories triggered by an odor, a taste, a sound, or a visual sensation? Because in everyday life people are often simultaneously exposed to combinations of sensory inputs from different modalities (e.g., the odor, taste, and texture of an apple), the relative power of the different senses to trigger autobiographical memories could be difficult to disentangle.

Among the five senses, the relationship between olfaction and memory has been at the heart of most studies in the field of cognitive neurosciences (for reviews, see Larsson et al., 2014; Saive et al., 2014; Hackländer et al., 2019). Research has suggested that odor-evoked memories have a particular status, which could be summarized under the acronym LOVER: *Limbic*, *Old*, *Vivid*, *Emotional*, and *Rare* (Larsson et al., 2014). Odor-evoked memories are associated with strong activations in the limbic and paralimbic cortices, as well as in the amygdala and the hippocampus (Herz et al., 2004; Arshamian et al., 2013). Another characteristic feature is that odors favor the retrieval of old childhood memories from the first decade of life (Chu and Downes, 2000; Willander and Larsson, 2006; Larsson and Willander, 2009; Miles and Berntsen, 2011), which contrasts with the typical reminiscence bump found in adolescence and early adulthood in autobiographical memory studies (Rubin et al., 1986; Rathbone et al., 2008; Koppel and Berntsen, 2015). With regard to their phenomenology, odor-cued memories are rated as more vivid and emotional than memories cued by other modalities (Chu and Downes, 2000; Larsson and Willander, 2009). Finally, studies have shown that odor cues produced fewer memories than verbal or visual cues and odor-evoked memories are thus considered as a relatively rare phenomenon (Chu and Downes, 2000; Goddard et al., 2005; Willander and Larsson, 2007).

Thus far, empirical studies have directly compared the influence of only two or three senses on various dimensions of autobiographical memory. Most previous research has examined the effect of olfactory and visual cues, and a couple of studies have also explored the influence of olfactory, visual, and auditory cues (e.g., Rubin et al., 1984; Chu and Downes, 2000; Goddard, et al., 2005; Willander and Larsson, 2006; Willander et al., 2015; Knez et al., 2017; de Bruijn and Bender, 2018). While these studies generally agree that odors outperform visual and/or auditory cues in terms of eliciting memories that are older and more emotional, more mixed results have been obtained for other memory features. For instance, it has been found that the type of sensory cues has either no differential effect on the episodic specificity and amount of details of autobiographical memories, or that odors have a negative influence on these dimensions (Herz et al., 2004; Goddard et al., 2005; Willander and Larsson, 2006, 2007). In the same vein, odor-evoked memories are generally less frequently rehearsed and less self-grounding than visual and/or auditory cues (Willander and Larsson, 2006, 2007; Knez et al., 2017).

This brief overview of the literature reveals an incomplete picture of the influence of the five senses on autobiographical memory. In particular, the extent at which gustatory and tactile sensations could act as powerful cues to trigger autobiographical memories remains somewhat mysterious, and yet these senses are ubiquitous in our everyday life. The memory of taste is an essential physiological function, which can impact physical and mental health, and even survival (Yamamoto and Yasoshima, 2007; Schiffman, 2009). Studies on conditioned taste aversion have shown that gustatory memories could be shaped even after a single exposure to the taste and could persist over the years (Yamamoto and Yasoshima, 2007; Chambers, 2015). Furthermore, there are many indications that the gustatory and olfactory chemosensory systems share a close functional relationship (Sugai et al., 2005; Doty, 2015). For instance, the presentation of an odor at a subthreshold concentration in conjunction with a subthreshold concentration of a taste enables the detection of the combination (Delwiche, 2004). In parallel, clinical studies have shown that olfactory impairment is associated with decreased taste function (Landis et al., 2010). Hence, one could expect that tastes, just like odors, would represent potent cues to trigger autobiographical memories, of which the phenomenological characteristics may approximate those of odor-evoked memories.

With respect to touch, tactile memory represents a relatively understudied research topic compared to other sensory modalities (Gallace and Spence, 2009). In fact, studies examining the interactions between memory and touch have mainly consisted of recognition memory tasks. Although the recognition of objects by sight is generally faster and more accurate, people’s ability to identify 3D objects or faces explored haptically is actually quite good and haptic memories can last for a lifetime (Klatzky et al., 1985; Gallace and Spence, 2009). However, beyond its role in exploring and identifying stimuli in our environment, touch has also important social and communicative functions, and tactile experiences could elicit strong emotional experiences (Hertenstein, 2002; Gallace and Spence, 2010). Hence, touch might be another important – and thus far overlooked – portal to autobiographical memory.

In this work, we thus ran the first comparative study examining the relative contribution of the five senses on the access, temporal distribution, rehearsal, and phenomenological characteristics (amount of details, personal importance, ease of retrieval, emotional valence, and intensity) of autobiographical memories. These dimensions all represent critical features of autobiographical memory (Rubin et al., 1986, 2003; Sutin and Robins, 2007). Our aim was 2-fold: (i) to replicate and extend previous research on visually-, auditory-, and odor-evoked memories in a large sample of adults from 18 to 80 years, and (ii) to test for the first time the influence of gustatory and tactile cues on autobiographical memory. Given the close relationship shared by olfaction and taste, we hypothesized that memories elicited by tastes would present similar characteristics to odor-evoked memories, that is, rare, old, and highly emotional memories. In parallel, we expected that visual and auditory cues would trigger more recent memories, which would be more specific, detailed and personally significant than memories cued by odors and tastes. Because little scientific research has been conducted on touch and memory, our approach was more exploratory here and we did not have specific hypotheses concerning the influence of tactile cues on autobiographical memories.

## Materials and Methods

### Memories and Participants

The participants were recruited *via* public engagement exercises conducted by the Universities of Bourgogne and Toulouse in 2015 and 2016 (“*La Nuit Européenne des Chercheurs*” and “*La Semaine du Cerveau*”). The study was performed in accordance with the ethical standards of the Declaration of Helsinki, and was approved by the organizing committees of the scientific events. As the experiment was conducted during public events, children and adolescents were also welcome to participate. However, due to ethical and methodological considerations, we included data only from participants aged over 18 years. A total of 2,627 responses to cues were generated, documented, and included in the study (see Table 1). Individual participants were not identifiable, but each participant wrote their age, gender, and level of education on each response sheet that they filled in and submitted for the research project. Each participant was free to generate between one and six autobiographical memories, and we estimate that ~400 participants took part in this project, based on the fact that almost all the participants completed all six conditions. As can be seen from Table 1, the mean age of participants did not differ according to the cue used to elicit the autobiographical memory and there were no differences in gender distribution and level of education according to the cue.

**Table 1:** Demographic characteristics of our groups of participants.

	All cues	Vision	Hearing	Touch	Taste	Olfaction	Words	Statistical analysis
*Age*								
Mean	30.85	31.63	30.61	31.01	30.64	30.84	30.58	*F*(5, 2,621) = 0.38, *p* = 0.86, $\eta_{p}^{2}$ = 0.86
SD	(13.11)	(13.30)	(12.74)	(13.27)	(13.04)	(13.06)	(13.26)	
[range]	[18–80]	[18–79]	[18–79]	[18–79]	[18–79]	[18–79]	[18–80]	
*Gender*								
Ratio female/male	1789/826	254/126	292/134	282/121	253/133	285/134	423/178	*χ^2^*(5, *N* = 2,615) = 3.47, *p* = 0.63
*Level of education*								
Ratio without/with a high school diploma	135/2489	23/356	24/402	23/384	16/375	22/398	27/574	*χ^2^*(5, *N* = 2,624) = 2.50, *p* = 0.78

Unlike standard approaches, the experimenters were also recruited, given that to run the event smoothly, we needed between 10 and 12 people, and at least seven people. These experimenters were familiarized with the protocol and were given a set of standard instructions to repeat at the different sites and events. They also were given materials to share in the debriefing session. These volunteers were also responsible for entering the data.

### Materials

Retrieval cues were selected from lists of cue-words used in previous studies (Crovitz and Schiffman, 1974; Zola-Morgan et al., 1983; Rubin et al., 2003), based on their familiarity, relevance, and suitability to be presented in a sensory form. We chose a total of ten cue-words among these lists: *house*, *cat*, *car*, *bird*, *cotton*, *sand*, *sugar*, *acid*, *flower*, and *tobacco*. From these words, we derived two sensory cues for each sense and these cue-words were also used as cues for a verbal control condition.

Visual cues (house and cat) were presented as drawings, which were mounted on white cards (measuring 21 cm × 29.7 cm). Sounds (a car starting and a singing bird) consisted of 10 s audio clips that were played through headphones. Textures (cotton and sand) were integrated into wrapped glass jars with a punctured lid to allow participants to touch the textures; participants wore a blindfold to ensure that the content was not visible. Tastes (sweet and sour) were obtained by diluting concentrates of tartaric acid or fructose in mineral water, with the following dosages: 0.61 g/L of tartaric acid and 7.39 g/L of fructose. During the testing session, participants received a half cup of solution to drink. Note that the two solutions were clear and visually identical, with no detectable odor. Olfactory cues (flower and tobacco) were presented in opaque jars containing a piece of baize on which two drops of ylang-ylang essential oil or three drops of tobacco synthetic aroma were dripped. The number of drops necessary to easily detect each odor, as well as the selection of ylang-ylang for the flower odor were determined in a pilot study with a dozen participants. After each presentation, the jars were immediately covered with a lid and their contents were replaced after each testing session to maintain odor quality and freshness. Finally, verbal cues from the control condition were presented as words, in capital letters, printed on white cards (measuring 21 cm × 29.7 cm). Critically, these verbal cues were the same stimuli as in each of the sensory conditions. That is, participants either could receive “tobacco” as an odor or as a cue word, and we arranged each testing session, such that participants could not receive a stimulus in the sensory condition as a control cue word.

Immediately after each retrieval attempt, the participants filled in a response sheet where they had to provide a brief title for the memory and their age at the time of the event. They also completed a series of rating scales assessing the specificity (unique vs. repetitive/routine) and the phenomenological characteristics of the retrieved event. More specifically, participants had to rate the emotional valence (*positive*, *neutral*, or *negative*) and intensity of the memory (1 = *not at all intense*, 4 = *very intense*), the amount of detail (1 = *very few details*, 4 = *very detailed*), ease of retrieval (1 = *very difficult*, 4 = *very easy*), and personal importance (1 = *very little important*, 4 = *very important*). Finally, participants indicated the last time when they had retrieved their memory (five response categories: *first retrieval*, *a few days ago*, *several weeks ago*, *several months ago*, or *several years ago*).

### Procedure

The experimental setting, named *the Proust Machine*, was organized in seven stands: five for the different sensory modalities and two for the verbal control condition. Participants were free to visit the stands and complete as many different conditions as they wanted during a 15-min testing session. Participants were instructed to provide the first specific autobiographical memory (i.e., a unique event that occurred at a particular time and place and lasted no longer than a day; an example was provided to ensure participants’ understanding of the notion of specificity) that came to their mind after the cue presentation. An experimenter was present at each stand to present the cue and collect the response sheet. If no memory was triggered by the retrieval cue, participants were asked to indicate it on the response sheet.

During each testing session, one cue per sensory modality (out of two) and two cues of the verbal control condition (out of ten) were presented. Within each condition, cues were pseudorandomly allocated across the multiple testing sessions, such that each cue was presented the same number of times in the whole experiment. As the verbal cues were derived from the sensory cues, as explained above, we also ensured that the same item was not presented in two different forms within the same testing session (i.e., if the picture of a house was presented for the visual condition, the word “house” could not be used in the verbal control condition).

At the end of the testing session, participants were invited to attend a popular science session on autobiographical memory in which the following topics were addressed: the existence of different memory systems, the identity function of autobiographical memory, the involuntary and voluntary modes of memory retrieval, the role of the senses in autobiographical memory, the reminiscence bump phenomenon, and finally, the brain network sustaining autobiographical memory.

## Results

A total of 2,627 responses were collected across all modalities. This included 380 responses elicited by visual cues, 426 triggered by sounds, 407 by textures, 392 by tastes, 420 by odors, and 602 by word-cues.^1^

Characteristics and properties of autobiographical memories across the six modalities were analyzed by means of one-way ANOVAs (with a significance level set at *p* < 0.01 with Bonferroni correction for multiple comparisons) or chi-square analyses for each dependent measure.

### Type of Information Generated

We examined first the influence of the different retrieval cues on the type of autobiographical information generated (unique, repetitive/routine, or absent) as classified by the participant. Our results showed that the type of information retrieved significantly differed according to the retrieval cue, *χ* ^2^(10, *N* = 2,627) = 253.98, *p* < 0.001. *Post hoc* comparisons were run to determine which sensory conditions were driving the statistically significant chi-square test, based on standardized residuals between observed and expected values (MacDonald and Gardner, 2000). Consistent with recommendations (MacDonald and Gardner, 2000; Sharpe, 2015), a Bonferroni correction for multiple comparisons was applied by dividing the alpha of 0.05 by 18 (i.e., the total number of cells in the chi-square test), which resulted in an adjusted *p* = 0.003. Table 2 shows that among visually-cued memories, the number of occasions where participants failed to retrieve a memory was particularly low. With respect to memories cued by a sound (hearing), we found that repetitive events were significantly overrepresented, whereas absent events was an underrepresented category of event. Among memories elicited by a taste, the number of specific and absent events were respectively, significantly lower and higher than the expected counts. Within odor-cued memories, all types of events deviated from the expected values: specific events and repetitive events were underrepresented and, absent events were overrepresented. Finally, the number of specific events elicited by a word was significantly higher than the expected count, whereas the number of absent events was particularly low in this condition.

**Table 2:** Number of memories generated for each type of event across the six conditions.

		Specific event	Repetitive event	Absence of event
Vision (*n* = 380)	Observed count	172	160	48
	Expected count	158.70	150.40	70.90
	Standardized residual	1.50	1.10	**−3.30**
	Probability value	0.13	0.28	**0.001**
Hearing (*n* = 426)	Observed count	166	224	36
	Expected count	177.90	168.60	79.50
	Standardized residual	−1.30	**6.00**	**−5.90**
	Probability value	0.20	**<0.001**	**<0.001**
Touch (*n* = 407)	Observed count	188	158	61
	Expected count	170.00	161.10	75.90
	Standardized residual	2.00	−0.30	−2.10
	Probability value	0.05	0.73	0.04
Taste (*n* = 392)	Observed count	128	134	130
	Expected count	163.70	155.20	73.10
	Standardized residual	**−4.00**	−2.40	**8.00**
	Probability value	**<0.001**	0.02	**<0.001**
Olfaction (*n* = 420)	Observed count	130	132	158
	Expected count	175.40	166.30	78.30
	Standardized residual	**−4.90**	**−3.70**	**10.90**
	Probability value	**<0.001**	**<0.001**	**<0.001**
Words (*n* = 602)	Observed count	313	232	57
	Expected count	251.40	238.30	112.30
	Standardized residual	**5.80**	−0.60	**−6.60**
	Probability value	**<0.001**	0.55	**<0.001**

Standardized residuals in bold are those that exceed ±1.96 (MacDonald and Gardner, 2000) and are significant using an adjusted *p* = 0.003 (Bonferroni correction).

### Phenomenological Characteristics of Autobiographical Memories

For the following, we analyzed the participant ratings and classifications of their own memories. Here we present the results for all valid responses. However, given that the phenomenological characteristics of memories are influenced by the episodic specificity of events, we also conducted the same set of statistical analyses but including only specific events. These restricted analyses produced the same patterns of results as below. The observed differences between sensory modalities were thus not simply due to differences in the proportions of specific and repetitive events.

#### Amount of Detail

**Figure 1:**
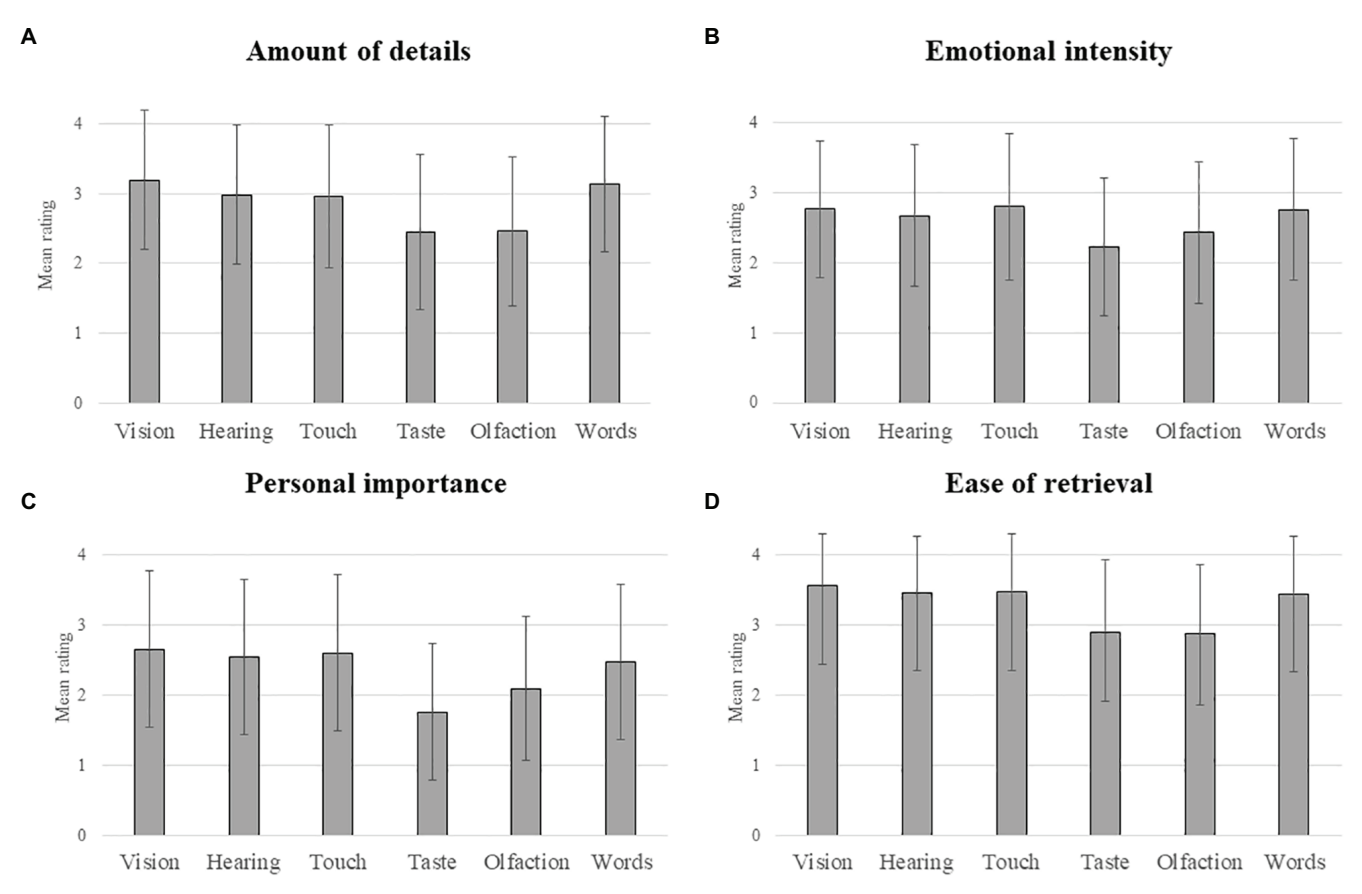
Mean ratings of the phenomenological characteristics of memories **(A)** amount of details; **(B)** emotional intensity; (C) personal importance; (D) ease of retrieval) elicited by the different types of cue. Error bars show the standard deviation.

The ANOVA on the subjective rating of the amount of detail revealed a significant influence of cue type, *F*(5, 2,129) = 32.07, *p* < 0.001, $\eta_{p}^{2}$ = 0.07 (Figure 1A). In particular, memories generated in response to both odors and tastes were less detailed than those triggered by pictures, sounds, textures, and words (*p* < 0.001 in every case). The remaining two-by-two comparisons did not yield significant results.

#### Emotional Valence

With respect to the emotional valence of autobiographical memories, we found that the number of positive, neutral, and negative events (as rated by participants) significantly varied across the six conditions, *χ* ^2^(10, *N* = 2,123) = 174.89, *p* < 0.001. Follow-up comparisons based on adjusted standardized residuals (Table 3; MacDonald and Gardner, 2000), with an adjusted *p* = 0.003 (Bonferroni correction for multiple comparisons), showed that in hearing and touch conditions, there were more positive and less negative memories than the expected count. In contrast, olfactory and gustatory conditions showed the opposite pattern, and we found a lower number of positive memories and a higher number of neutral and negative memories.

**Table 3:** Number of positive, neutral and negative memories generated across the six conditions.

		Positive	Neutral	Negative
Vision (*n* = 331)	Observed count	213	71	47
	Expected count	194.10	86.20	50.70
	Standardized residual	2.30	−2.10	−0.60
	Probability value	0.02	0.04	0.54
Hearing (*n* = 387)	Observed count	257	106	24
	Expected count	227	100.80	59.20
	Standardized residual	**3.40**	0.70	**−5.50**
	Probability value	**0.001**	0.51	**<0.001**
Touch (*n* = 345)	Observed count	254	74	17
	Expected count	202.30	89.90	52.80
	Standardized residual	**6.20**	−2.10	**−5.90**
	Probability value	**<0.001**	0.03	**<0.001**
Taste (*n* = 260)	Observed count	91	90	79
	Expected count	152.50	67.70	39.80
	Standardized residual	**−8.30**	**3.40**	**7.20**
	Probability value	**<0.001**	**0.001**	**<0.001**
Olfaction (*n* = 261)	Observed count	112	92	57
	Expected count	153.10	68	40
	Standardized residual	**−5.50**	**3.60**	**3.10**
	Probability value	**<0.001**	**<0.001**	**0.002**
Words (*n* = 539)	Observed count	318	120	101
	Expected count	316.10	140.40	82.50
	Standardized residual	0.20	−2.30	2.60
	Probability value	0.85	0.02	0.01

Standardized residuals in bold are those that exceed ±1.96 (MacDonald and Gardner, 2000) and are significant using an adjusted *p* = 0.003 (Bonferroni correction).

#### Emotional Intensity

The type of retrieval cue also has a significant influence on the emotional intensity of memories, *F*(5, 2,127) = 15.02, *p* < 0.001, $\eta_{p}^{2}$ = 0.03 (Figure 1B). *Post hoc* comparisons indicated that memories triggered by tastes were less emotionally intense than those evoked by visual and auditory cues, textures, or words (*p* < 0.001 in every case). In the same vein, olfactory cues elicited less emotionally intense memories than visual cues (*p* = 0.002), textures (*p* < 0.001), and words (*p* = 0.003). No other comparison reached statistical significance.

#### Personal Importance

Our findings showed that the personal importance of autobiographical memories varied as a function of the type of retrieval cue, *F*(5, 2,128) = 30.04, *p* < 0.001, $\eta_{p}^{2}$ = 0.07 (Figure 1C). Autobiographical memories triggered by tastes were rated as less personally important than the five other types of cues (*p* < 0.001 for pictures, sounds, textures, and words and *p* = 0.006 for odors). Furthermore, ratings of personal importance were also lower for odor-evoked memories than for memories elicited by visual and auditory cues, textures, and words (*p* < 0.001 in every case). No other comparison was significant.

#### Ease of Retrieval

The perceived ease with which participants retrieved memories was also significantly influenced by the type of retrieval cue used to trigger memories, *F*(5, 2,126) = 38.52, *p* < 0.001, $\eta_{p}^{2}$ = 0.08 (Figure 1D). Participants experienced more difficulty in retrieving autobiographical memories in response to both odors and tastes than after having been exposed to pictures, sounds, textures, or words (*p* < 0.001 in every case). No other statistical comparison was significant.

#### Effect of Gender on the Phenomenological Characteristics of Memories

For an exploratory purpose, we also examine whether the phenomenological characteristics of memories evoked by the different sensory cues varied across gender. We found no significant main effect of gender on the amount of detail [*F*(1, 2,114) = 3.56, *p* = 0.06, $\eta_{p}^{2}$ = 0.002], the emotional intensity [*F*(1, 2,112) = 0.81, *p* = 0.37, $\eta_{p}^{2}$ = 0.0004], or the ease of retrieval of memories [*F*(1, 2,111) = 1.63, *p* = 0.20, $\eta_{p}^{2}$ = 0.0008]. In addition, no significant interactions between gender and the type of sensory cues were found in these dimensions [amount of detail: *F*(5, 2,114) = 2.07, *p* = 0.07, $\eta_{p}^{2}$ = 0.07; emotional intensity: *F*(5, 2112) = 2.27, *p* = 0.05, $\eta_{p}^{2}$ = 0.005; and ease of retrieval: *F*(5, 2,111) = 2.16, *p* = 0.06, $\eta_{p}^{2}$ = 0.005]. However, a significant effect of gender was found for the personal importance of memories, showing that women rated their memories as more personally significant than men, *F*(1, 2,113) = 13.62, *p* < 0.001, $\eta_{p}^{2}$ = 0.006 (women: mean score = 2.46, *SD* = 1.13; men: mean score = 2.26, *SD* = 1.09). A significant interaction between gender and the type of sensory cues was also found, *F*(5, 2,113) = 3.35, *p* = 0.005, $\eta_{p}^{2}$ = 0.008. *Post hoc* analyses showed that the personal importance of memories elicited by a visual cue was significantly higher in women than in men (*p* = 0.004). The remaining two-by-two comparisons were not statistically significant (all *p* > 0.61).

### Time Elapsed Since Last Retrieval

We were also interested in the time elapsed between the last time the participant retrieved a memory and its retrieval at the time of the study. Our results showed that this time window significantly varied across the six types of memory cue, *χ* ^2^(20, *N* = 2,120) = 105.36, *p* < 0.001. Follow-up comparisons using adjusted standardized residuals (MacDonald and Gardner, 2000) and an adjusted *p* = 0.002 (Bonferroni correction for multiple comparisons) showed that in the vision condition, there was a lower number of memories recalled for the first time, and a higher number of memories rehearsed within the last few days. In addition, among memories elicited by taste, we found a greater number of memories retrieved for the first time and a lower number of memories rehearsed within the last few days. A similar profile was found for odor-cued memories, although the first time category did not survive the statistical threshold corrected for multiple comparisons. Finally, there was a greater number of memories evoked within the last few days in the control condition (words; Table 4).

**Table 4:** Number of memories as a function of the time elapsed since their last rehearsal across the six conditions.

		First time	Days	Weeks	Months	Years
Vision (*n* = 328)	Observed count	61	82	43	60	82
	Expected count	91.60	54.80	40.20	57.90	83.50
	Standardized residual	**−4.10**	**4.40**	0.50	0.30	−0.20
	Probability value	**<0.001**	**<0.001**	0.61	0.74	0.83
Hearing (*n* = 386)	Observed count	109	66	50	72	89
	Expected count	107.80	64.50	47.30	68.10	98.30
	Standardized residual	0.20	0.20	0.50	0.60	−1.20
	Probability value	0.88	0.82	0.65	0.56	0.23
Touch (*n* = 345)	Observed count	93	40	55	66	91
	Expected count	96.30	57.60	42.30	60.90	87.90
	Standardized residual	−0.40	−2.80	2.30	0.80	0.40
	Probability value	0.66	0.005	0.02	0.43	0.67
Taste (*n* = 261)	Observed count	110	24	23	33	71
	Expected count	72.90	43.60	32	46	66.50
	Standardized residual	**5.50**	**−3.50**	−1.80	−2.30	0.70
	Probability value	**<0.001**	**0.001**	0.07	0.02	0.49
Olfaction (*n* = 262)	Observed count	93	24	22	41	82
	Expected count	73.20	43.70	32.10	46.20	66.70
	Standardized residual	2.90	**−3.50**	−2.00	−0.90	2.30
	Probability value	0.004	**<0.001**	0.04	0.37	0.02
Words (*n* = 538)	Observed count	126	118	67	102	125
	Expected count	150.20	89.80	66	94.90	137
	Standardized residual	−2.70	**3.80**	0.20	0.90	−1.40
	Probability value	0.007	**<0.001**	0.88	0.35	0.17

Standardized residuals in bold are those that exceed ±1.96 (MacDonald and Gardner, 2000) and are significant using an adjusted *p* = 0.002 (Bonferroni correction).

### Temporal Distribution of Memories

**Figure 2:**
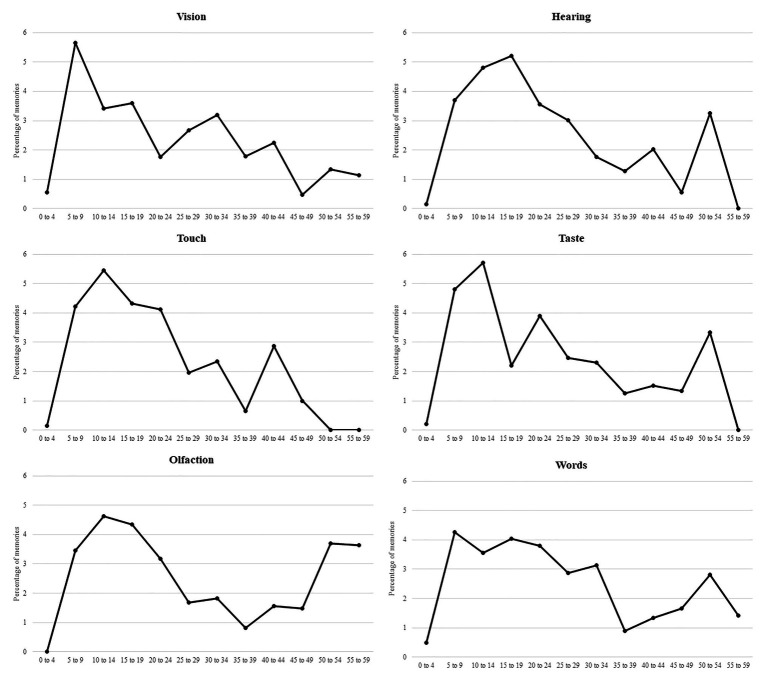
Lifespan retrieval curves for each type of cue.

The mean age at encoding for memories did not vary significantly across the six types of memory cue, *F*(5, 1,962) = 1.52, *p* = 0.18, $\eta_{p}^{2}$ = 0.004. To examine the distribution of memories, we calculated separate lifespan retrieval curves for each type of cue (see Figure 2). The lifespan retrieval curve plots the percentage of memories generated for each age bin independently. We calculated the number of memories recalled and we then used the number of participants in each age bin to calculate a percentage, such that if 149 people in our sample were aged 50–55, and they generated only two memories in this same bin, the percentage would be 1.34, i.e., ~1% of the sample at this age interval. This method controls for the fact that the distribution of ages itself could lead to an artifactual reminiscence bump (i.e., because so many of our sample are aged 18–30, it is not logically possible to have many memories which are *not* in the reminiscence bump period). In this analysis, we deleted bins where there were fewer than 10 participants for anyone specific age. In effect, this resulted in having no data beyond the age of 60. A qualitative analysis of the retrieval curves shows that, on the whole, the temporal distribution of memories followed the same profile across the five senses. In particular, we found an early reminiscence bump between 5 and 19 years in the vision, hearing, touch, taste, and olfaction conditions. However, the reminiscence bump in the control condition (words) appeared wider and spread between 5 and 34 years.

**Figure 3:**
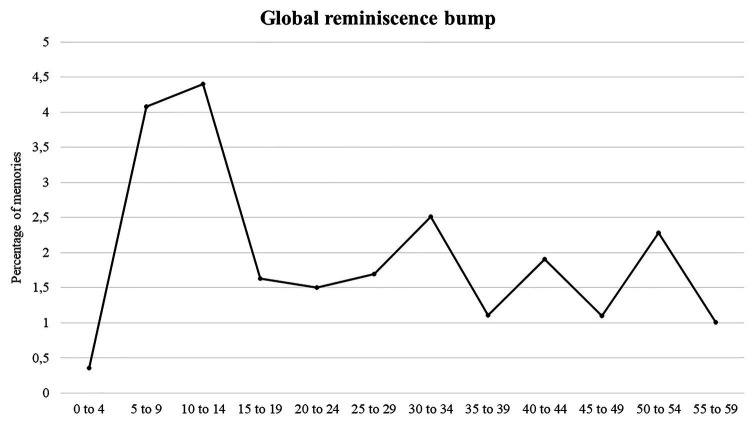
Global reminiscence bump with the six conditions grouped together in participants aged over 30.

To be satisfied that the reminiscence bump was produced even without our large numbers of younger participants, we also calculated a reminiscence bump where all six conditions were grouped together and participants under the age of 30 were removed. The standard reminiscence bump obtained (using the same calculation procedure) is shown in Figure 3.

## Discussion

Our senses are constantly stimulated in our daily lives but we have only limited a understanding of how they affect our cognitive processes and, in particular, our autobiographical memory. The retrieval of a sometimes long-forgotten memory triggered by a tiny sensory stimulus in the environment is a puzzling experience, which has inspired numerous writers and artists (van Campen, 2014), but this phenomenon has been relatively underexplored in the field of cognitive sciences. Here, capitalizing on a public science event, we conducted the first empirical study that aimed to directly compare the relative influence of the five senses on the access, temporal distribution, and phenomenological characteristics of autobiographical memories in a large sample. Our results demonstrated that the type of sensory cues modulates the access to memories and their phenomenological features. By and large, visual, auditory, tactile, and verbal cues outperformed olfactory and gustatory cues in eliciting specific, detailed, emotional, and personally important memories. However, odors and tastes appeared particularly powerful to trigger memories that had not been evoked by the participants before the testing session. We found that the temporal distribution of memories followed the same profile across the five senses.

An important contribution of this study is to show for the first time that gustatory- and odor-evoked autobiographical memories present largely similar characteristics. These shared features may be the result of the strong interactions between these two chemosensory systems, which are most of the time stimulated simultaneously in daily life activities such as eating or drinking (Delwiche, 2004; Landis et al., 2010). More specifically, our results partially replicated previous findings on the distinguishing features of odor-cued memories and extended them to gustatory-evoked memories. Consistent with previous studies (Chu and Downes, 2000; Goddard et al., 2005; Larsson et al., 2014), we found that odor- and gustatory-evoked memories are rare phenomena: odors and tastes produced fewer memories, which were also judged as more difficult to retrieve, and less specific and unique than memories from the other categories of sensory cues. In addition, olfactory and gustatory cues also triggered less detailed and less personally important autobiographical memories (see Goddard et al., 2005; Knez et al., 2017 for similar results on odor-evoked memories). Interestingly, however, we determined for the first time that odors and tastes tended towards “digging out” buried memories that had not been evoked before the testing session. Memory models such the Self-Memory System (SMS; Conway and Pleydell-Pearce, 2000; Conway, 2005) predict that, over time, autobiographical memories that have low self-relevance are unlikely to be frequently rehearsed, which attenuates, in turn, their accessibility and episodic quality. However, a central postulate of the SMS is also that a potent cue corresponding to some component of the past experience might overcome lowered accessibility to an apparently forgotten (but still available) memory. Odor- and gustatory-evoked memories appear to illustrate well this theoretical assumption and make a valuable case for the distinction between the accessibility and availability of past event representations in memory (Tulving and Pearlstone, 1966; Conway, 2009). It is perhaps this unpredictable and oblique cueing action for odor- and gustatory-stimuli which has led to the characterization of odor cued memories as being poignant, and particularly evocative.

Unexpectedly, we only partially replicated the finding that odors cued more emotionally charged memories. We found that odors and tastes elicited less positive but more neutral and negative memories, which were also rated as less emotionally intense than memories triggered by the other sensory cues. In the same vein, while we replicated the finding that odors favor the retrieval of childhood memories (Chu and Downes, 2000; Willander and Larsson, 2006; Larsson and Willander, 2009), this early reminiscence bump appears here wider than in previous studies as it spreads until early adulthood (15–19 years). Most importantly and contrary to previous studies (see Larsson et al., 2014 for a review), we found that the general temporal distribution of memories followed globally the same trajectory across the five senses and the presence of an early reminiscence bump was not a distinctive feature of odor-evoked memories. A critical aspect to account for these results may concern the selection of sensory cues. Recently, de Bruijn and Bender (2018) have demonstrated that the use of odors associated with childhood is determining to trigger memories from this particular life period. In other words, a high congruency between the cue and the memory may be an important (if not necessary) factor to favor the retrieval of old childhood memories. One could speculate that this congruency effect may extend beyond the specific case of odor-evoked memories and that all our sensory cues were particularly prone to trigger childhood memories (as a reminder, the list of cue-words from which we derived the sensory cues was: *house*, *cat*, *car*, *bird*, *cotton*, *sand*, *sugar*, *acid*, *flower*, and *tobacco*). We, however, acknowledge that this remains speculative as we did not collect any information about the privileged association of our cues with this life period. Further studies controlling for the temporal matching of sensory cues with specific life periods would refine our understanding on the proneness of each sense to elicit memories from particular life periods and elucidate whether this congruency effect is unique to odor-evoked childhood memories or is a more general phenomenon.

Among the five senses, the vision has often been found as having an advantage over other senses in humans in a variety of cognitive domains (Sinnett et al., 2007; Schmid et al., 2011). In this regard, autobiographical memory is not an exception: visual imagery processes play a central role in the retrieval and detail elaboration of memories by activating multisensory information in a cascading fashion (Greenberg and Rubin, 2003; Ernst et al., 2015). Several studies have shown that when participants are exposed to multimodal cues (consisting for instance in combinations of pictures, sounds, and odors), the retrieval and content of autobiographical memories are primarily driven by the visual modality (Karlsson et al., 2013; Willander et al., 2015). Furthermore, according to these studies, the second most significant modality in the sensory hierarchy is the auditory system. Our findings support this view by showing that visual and auditory cues outperformed odors and tastes to elicit specific, detailed, emotional, and personally significant memories. However, we did not find evidence in favor of a hierarchy between the visual and auditory systems as both types of cues had a similar influence on these phenomenological characteristics. Qualitatively, we only observed that the age distribution of visually cued memories showed an earlier and narrower peak (5–9 years) than auditory-evoked memories (from 5–9 to 20–24 years).

Thus far, our results suggest that a certain degree of overlap may exist between some senses with regard to their influence on autobiographical memory, with on one hand, olfaction and taste and, on the other, vision and audition. In this grouping, our findings also suggested for the first time that touch approximates vision and audition. Indeed, we found that tactile cues are just as powerful as visual and auditory information to elicit autobiographical memories, which present highly similar phenomenological features. Furthermore, we found that auditory and tactile cues triggered more positive memories and less negative ones than the other types of sensory cues. Striking similarities were also observed between the temporal distribution of memories cued by sounds and textures: both showed a reminiscence bump that spreads from 5–9 to 20–24 years. This pattern of results thus raises new questions about the role of touch in human memory and its relationship with vision and audition. Neuroimaging studies have shown that visual cortical areas are involved in haptic object recognition, which likely reflects the formation of mental images during haptic exploration (see Gadelha et al., 2013 for a review). Although this hypothesis remains to be investigated in detail, we suggest that this neurofunctional convergence may account, at least in part, for the similarities observed between autobiographical memories elicited by visual and tactile cues.

Since gender differences in autobiographical memory have frequently been reported in the literature (for a review see Grysman and Hudson, 2013), we also conducted an exploratory analysis to examine whether the phenomenological characteristics of memories evoked by the different sensory cues varied across gender. Previous studies have shown that, when gender differences are reported, women’s autobiographical memories are generally rated as more emotional, vivid, and personally significant (Grysman and Hudson, 2013). In our large sample of participants, we partially replicated these findings by showing that women rated their memories as more personally meaningful than men, especially for visually-cued memories. Among the different autobiographical memory studies using the sensory cue approach, the effect of gender has been rarely explored. To our knowledge, Goddard et al. (2005) were the only to address this issue and they found no gender differences, except that women rated their memories as being more vivid than men. Future studies addressing more specifically this issue are thus needed, especially since the expression of gender differences is influenced, among other dimensions, by the methodology used to elicit autobiographical memories (Grysman and Hudson, 2013).

Taken together, the current findings refine our understanding of the influence of the senses on autobiographical memory functioning and organization. Our results show that sensory information can act as powerful cues to trigger memories, of which the phenomenological and qualitative properties depend on the nature of the sensory cue. Of particular interest, this comparative study suggests that, within the five senses, some of them may share a particular relationship as they have a similar influence on autobiographical memory: on one side lie olfaction and taste, and on the other vision, audition and touch. This finding raises new questions about the contribution of multisensory processing to autobiographical memory. Indeed, while this study was designed to investigate the respective influence of each sense, a more ecological approach would be to examine the effect of multimodal cues on the elicitation of memories, as multiple senses are generally stimulated simultaneously in everyday life. Previous studies along this line have shown a positive influence of multimodal cueing on autobiographical recall when it combined visual, auditory, and olfactory cues (Karlsson et al., 2013; Willander et al., 2015). Interestingly, both studies have shown that autobiographical recall was primarily driven by the visual and auditory modalities, and to a lesser extent by olfactory cues. As such, the sensory organization suggested by our findings may offer new lines of inquiry to better understand the impact of multimodal cueing on autobiographical memory and to delve deeper into the cognitive and sensory mechanisms that support this organization. In particular, despite its central role in autobiographical memory (Greenberg and Rubin, 2003; Ernst et al., 2015), in the current study, we did not assess the visual imagery vividness of memories and yet, visual imagery processes appear as a good candidate to explain, at least in part, the similarities between vision and touch. Future studies should examine in more detail the contribution of visual imagery processes in the differential influence of sensory cues on autobiographical memory. Despite these advances in the understanding of the Proust effect, one has to acknowledge that, by its very nature, this phenomenon is difficult to capture with empirical testing for at least two reasons. First, in its original description, the unexpected and involuntary recall of a memory is at the heart of this phenomenon, which clearly contrasts with experimental paradigms asking participants to generate deliberately memories in response to sensory cues. Another important aspect relates to the fact that sensory-memory associations are different and unique for every individual, which makes the use of standardized sensory cues ill-suited. In this regard, the use of personalized cues might be a more naturalistic approach (see de Bruijn and Bender, 2018 for further discussion on this issue). Future studies developing innovative study designs may overstep these limitations to get closer to the original phenomenon experienced by people in daily life.

Studies along this line could also stimulate applied research in clinical psychology, and especially the development of reminiscence therapies (Duru Aşiret and Kapucu, 2016; Glachet et al., 2018; Mileski et al., 2018; Kirk et al., 2019). Among the different approaches, the use of sensory-based reminiscence therapy appears particularly promising since sensory cues could provide a more direct access to autobiographical memories and thus minimize the demand on executive control processes (Kirk and Berntsen, 2018; Glachet and El Haj, 2019). For instance, the use of odors or music cues have been found to enhance the specificity and the phenomenological experience of memories evoked by people with Alzheimer’s disease (El Haj et al., 2012; Glachet and El Haj, 2019), and odors also improved the access to self-concepts (Glachet and El Haj, 2020). These previous results thus contrast with some of our current findings in healthy subjects. This suggests that the influence of odors on autobiographical memory might be modulated by the preservation/impairment of different cognitive mechanisms engaged in autobiographical retrieval, in particular, higher-order executive functions and strategic processing that are known to be involved in autobiographical memory decline in Alzheimer’s disease (for a review see El Haj et al., 2015). Furthermore, Kirk et al. (2019) have shown that the use of an immersive setting consisting in an apartment that matches the time of participants’ youth and which stimulates different sensory modalities (i.e., vision, olfaction, audition, somatic sensation) significantly improve the access to episodic autobiographical memories in people with Alzheimer’s disease. While most studies to date have been conducted in people with dementia, the extension of sensory-based reminiscence therapy to psychopathology might represent a future research avenue. Indeed, a recent study has shown that olfactory autobiographical memories were associated with higher levels of subjective feeling of belongingness in people with schizophrenia (Allé et al., 2020). Stimulating autobiographical memory by odors might thus be a way to alleviate the executive-related autobiographical memory deficit observed in schizophrenia and it might also have a positive influence on some associated pathological features such as the reduced sense of self (Berna et al., 2016). However, despite the great popularity and potential of reminiscence therapy, to date, there is no golden standard regarding the method and procedure to apply. More empirical studies comparing the properties of autobiographical memories across the five senses are thus needed, including in clinical groups, to determine the most suitable memory triggers and how to use them to maximize the effect on autobiographical remembering.

As a final point, it is also worth discussing some advantages and limitations of the public event method. There is a growing movement to have larger sample sizes and more generalizable results in experimental psychology, leading to the use of online testing where the demographic characteristics are more varied than the typical undergraduate sample, and the sample sizes are less constrained by financial and practical considerations. However, online testing has its constraints: the lack of use of tactile, gustatory, and olfactory stimuli being notable. Here, we want to acknowledge several constraints, which are inevitable in using large public events, such as the possible influence of a social environment, background noise, lack of tight control on within-subject manipulations (as was the case here) as a result of promoting a flexible and friendly format, little or no checking for the understanding of instructions or verification of responses and the undoubted high motivation of a self-selected sample.

Our over-riding conclusions are positive, however, since with such large-scale studies, we can test the generalizability and robustness of effects and test hypotheses in novel groups. Here, for instance, our data produce patterns, which are in keeping with known findings in more tightly controlled experiments. Our effect sizes are notably small, but the study has yielded new insights into autobiographical memories cued by smells, tastes, and touch in comparison with other more usual auditory, verbal, and visual cues. These exploratory findings will need verification in standard laboratory tasks, but for now, it is thanks to the enthusiasm and interest of our public event participants and experimenters that these new insights have come to light.

## Data Availability Statement

The datasets presented in this study can be found in online repositories. The names of the repository/repositories and accession number(s) can be found at: https://osf.io/qv2xb/?view_only=e3628519613748509f794be48934b6ef.

## Ethics Statement

Ethical review and approval was not required for the study on human participants in accordance with the local legislation and institutional requirements. Written informed consent for participation was not required for this study in accordance with the national legislation and the institutional requirements.

## Author Contributions

AE: conceptualization, methodology, formal analysis, and writing. JB: conceptualization, methodology, investigation, and formal analysis. VV: methodology and investigation. CS: conceptualization, methodology, resources, supervision, review, and editing. CM: conceptualization, methodology, resources, formal analysis, supervision, writing, review, and editing. All authors contributed to the article and approved the submitted version.

### Conflict of Interest

The authors declare that the research was conducted in the absence of any commercial or financial relationships that could be construed as a potential conflict of interest.

## Footnotes

^1^The raw data are freely available on the Open Science Framework: https://osf.io/qv2xb/?view_only=e3628519613748509f794be48934b6ef.
